# Construction of five cuproptosis-related lncRNA signature for predicting prognosis and immune activity in skin cutaneous melanoma

**DOI:** 10.3389/fgene.2022.972899

**Published:** 2022-09-07

**Authors:** Xiaojing Yang, Xing Wang, Xinti Sun, Meng Xiao, Liyun Fan, Yunwei Su, Lu Xue, Suju Luo, Shuping Hou, Huiping Wang

**Affiliations:** ^1^ Department of Dermatovenereology, Tianjin Medical University General Hospital, Tianjin, China; ^2^ Department of Thoracic Surgery, Tianjin Medical University General Hospital, Tianjin, China

**Keywords:** skin cutaneous melanoma, lncRNA, cuproptosis, immune therapy, bioinformatics

## Abstract

Cuproptosis is a newly discovered new mechanism of programmed cell death, and its unique pathway to regulate cell death is thought to have a unique role in understanding cancer progression and guiding cancer therapy. However, this regulation has not been studied in SKCM at present. In this study, data on Skin Cutaneous Melanoma (SKCM) patients were downloaded from the TCGA database. We screened the genes related to cuproptosis from the published papers and confirmed the lncRNAs related to them. We applied Univariate/multivariate and LASSO Cox regression algorithms, and finally identified 5 cuproptosis-related lncRNAs for constructing prognosis prediction models (VIM-AS1, AC012443.2, MALINC1, AL354696.2, HSD11B1-AS1). The reliability and validity test of the model indicated that the model could well distinguish the prognosis and survival of SKCM patients. Next, immune microenvironment, immunotherapy analysis, and functional enrichment analysis were also performed. In conclusion, this study is the first analysis based on cuproptosis-related lncRNAs in SKCM and aims to open up new directions for SKCM therapy.

## Introduction

Skin cutaneous melanoma (SKCM) is the most aggressive skin cancer with high mortality and rapid metastatic potential (Rodriguez-Hernandez et al., 2020). Global cancer statistical analysis shows significant increases in morbidity and mortality of SKCM in recent years (Schadendorf et al., 2018; Sung et al., 2021). However, even though SKCM only accounts for 5% of all malignant tumors of the skin, it is responsible for 75% of the deaths associated with cutaneous neoplasms (Siegel et al., 2021). For localized or regional melanoma, first-line treatment remains surgical resection and 5-years survival rates are 98% and 64%. Due to chemotherapy resistance and aggressive clinical behavior of advanced melanoma, the 5-years survival rate is only 23% (Rebecca et al., 2020). Considering that traditional treatments have been the main treatment for advanced melanoma in long term, it only relieves some symptoms and reduces the tumor burden, which does not help prolong survival. Therefore, novel effective biomarkers and risk modeling of SKCM are necessary for improving early diagnosis, predicting prognosis, and guiding clinical treatment.

Recently, a study published in the journal *Science* first reveal that cuproptosis, as a new type of programmed cell death (PCD), differs from previous PCD such as ferroptosis, apoptosis, and autophagy in its special mechanism. Cuproptosis is mediated by a copper-sulfur protein, where copper binds to lipid acylation in the TCA cycle, resulting in the aggregation of lipid acylated proteins and subsequent loss of iron-sulfur cluster proteins, resulting in proteotoxic stress and ultimately cell death (Tsvetkov et al., 2022). Jiang et al. (2022a) have discovered that cuproptosis induces tumor cell death by activating apoptosis pathways by creating reactive oxygen species (ROS), opening a new avenue for anti-cancer research. Besides, copper was found to be accumulating in serum samples from patients with cancer, indicating copper may play a significant role in cancer progression (Ebara et al., 2000; Zabłocka-Słowińska et al., 2018; Feng et al., 2020). Besides, Chen et al. (2019) confirmed that an anti-tumor agent of copper-dependent can exert an effective anti-tumor effect on the hematopoietic system *in vivo*/vitro experiments. Bian et al. (2022) constructed a novel cuproptosis-related signature to predict prognosis and provide new insights into therapeutic strategies in clear cell renal cell carcinoma.

Long non-coding RNAs (lncRNAs) are non-coding transcripts with 200 nucleotides in length, which have been shown to regulate the expression of cancer-related genes (Alexander et al., 2010). In recent studies, it was determined that changes in the expression and function of lncRNA might be closely related to PCDs such as apoptosis, autophagy, and ferroptosis (Jiang et al., 2021; Qi et al., 2022a). For example, LncRNA RP11-89 was confirmed to be a novel tumorigenic modulator that inhibits ferroptosis through PROM2-activated iron export and might serve as one of the biomarkers to guide targeted therapy for bladder cancer (Luo et al., 2021). Besides, Shen et al. identified 10 N6-methyladenosine (m6A)-related lncRNAs as significantly related to the prognosis of SKCM and further constructed a risk model by using bioinformatics analysis (Shen et al., 2022). There is, however, no knowledge of the role of lncRNAs in cuproptosis of the SKCM, the latest results of Haozhen Lv et al. showed that the three Cuproptosis-related genes, LIPT1, PDHA1, and SLC31A1, have a predictive effect on the prognosis of SKCM patients, which also gave us a hint for further research to a certain extent (Lv et al., 2022). So, we decided to use bioinformatics analysis to uncover the relationship between cuproptosis, lncRNA, and SKCM.

In addition, immunotherapy has recently attracted much attention as a new tumor treatment strategy. The study of Elena Gómez-Abenza et al. in zebrafish pointed out that changes in the SKCM cellular immune microenvironment (TME) can be regulated by SPINT1 (Gómez-Abenza et al., 2019), while Min Yan 1 et al. (Yan et al., 2021), based on the results of single-cell sequencing, specifically pointed out that T cells in SKCM unique role. Therefore, in this study, we also intend to explore the immune system changes in SKCM patients.

In this study, based on the TCGA database, an accurate prognostic model for SKCM was constructed, and multiple cuproptosis-related lncRNAs were identified as potential biomarkers. Furthermore, we carry out a comprehensive analysis of the risk model including functional enrichment, drug resistance, immunotherapy, immune infiltration, and somatic mutation. Hopefully, the findings of our study will provide insight into the role of cuproptosis-related lncRNAs in SKCM.

## Materials and methods

### Data collection

From the TCGA database (http://portal.gdc.cancer.gov/), RNA sequencing data, somatic mutations, as well as the corresponding clinical information of SKCM samples were obtained. To ensure the authenticity of the analysis results as much as possible, we removed samples with no survival time and survival times less than 30 days. Ultimately, 455 patients of the SKCM were included in the analyses; they were divided randomly among training (*n* = 228) and testing sets (*n* = 227) using the R package “caret”. The Chi-square tests were applied to compare the clinical characters between the training and testing sets. Cuproptosis regulators (Supplementary Table S1) were obtained from previous literature (Tsvetkov et al., 2022).

### Identification of cuproptosis-related lncRNAs

We obtained 16773 lncRNAs according to the annotation file of lncRNA obtained from the GENCODE database (https://www.gencodegenes.org/human/). Pearson correlation test was further conducted to identify cuproptosis-related lncRNAs following the filter criteria (|R| ≥ 0.4 and *p-*value < 0.001).

### Construction of cuproptosis-related risk model

First, we identified 142 prognostic cuproptosis-related lncRNAs using Univariate Cox regression analysis. Furthermore, LASSO regression analysis (Lupton-Smith et al., 2021) was used to prevent overfitting, and 9 cuproptosis-related lncRNAs were identified to be significantly associated with overall survival (OS) in SKCM patients. Finally, to identify the powerful candidate lncRNAs and establish the risk model, we conducted a multivariate Cox regression analysis and 5 cuproptosis-related lncRNAs were considered prognostic factors. Use the formula below to calculate the risk score (It is worth noting that *coef* is an abbreviation for the corresponding coefficient, and *Exp* is an abbreviation for lncRNA’s expression):

**Risk score** = ∑Coef lncRNAs×Exp lncRNAs

We calculated the risk score for each SKCM patient using the formula and further used the median as a cutoff to subgroup the patients (high-risk group and low-risk group).

### Assessment of the prediction accuracy of risk model

We conducted Kaplan-Meier (K-M) analysis to assess the risk model prediction ability using the R package “survival”. Besides, the Receiver operating characteristics (ROC) curve of 1-, 3-, and 5-years was drawn to further verify the predictive power of the established risk prognostic model using the package “timeROC”. Performed principal component (PCA), as well as t-distributed stochastic neighbor embedding (t-SNE) analyses, were applied to lessen the dimensions and visualize the distinction between the two groups.

### Independence of the risk model

We conducted univariate regression, and multivariate regression analysis to verify whether our risk model can predict the prognosis of SKCM patients independently of other clinical factors (Gender, Age, Pathological stage, and TNM stage).

### Establishment of the nomogram

Studies have shown that Nomogram can accurately calculate the survival rate of tumor patients and has great value in clinical applications (Awan et al., 2021). We further applied the R package “rms” to build a nomogram, combining a variety of key clinical factors and risk models to better predict long-term survival in SKCM patients. To verify that the actual results and model predictions are in agreement, a calibration plot was drawn using the Hosmer-Lemeshow test.

### Analysis of immune microenvironment and molecular variation

We utilized the ESTIMATE algorithm to calculate the immune, stromal, and estimate scores to assess the differences in tumor microenvironments (TMEs) between two groups (Yu et al., 2021). Besides, we assess the levels of immune cells of entire SKCM patients using the CIBERSORT algorithm (Guan et al., 2022). Furthermore, we applied ssGSEA and GSVA analyses to explore the discrepancy between infiltrating fractions of immune cells and immune-related functions between the two groups (Zhang et al., 2021a; Xu et al., 2022). We analyzed tumor mutation burden (TMB) using the package “maftools” and divided all SKCM patients into high- and low-TMB groups according to the median TMB score. Besides, we calculated the correlation between the risk model and TMB using Pearson correlation analysis.

### The therapeutic significance of the risk model

To better apply the model to clinical treatment, we calculated the IC50 values of common anti-SKCM drugs by using the R package “pRRophetic” (Geeleher et al., 2014). Furthermore, to identify potential drugs that can treat SKCM, we identified many compounds obtained from the GDSC website (https://www.cancerrxgene.org/) with significantly different IC50 values between the two groups. To investigate the potential benefits of the risk model in immunotherapy, we also compared the expression levels of critical immune checkpoint genes (ICIs), including PD-1, PD-L1, HAVCR2, and CTLA-4, between two groups.

### Functional enrichment analysis

Differentially expressed genes (DEGs) between two groups were identified by using the package “limma” following the criteria (|Log2FC| > 1.0, *p-*value < 0.05). We further applied GO and KEGG functional enrichment analyses to investigate the related functions and pathways on the bias of the DEGs using the package “clusterProfiler”. We further conducted a GSEA analysis to compare the potential pathways between two groups using GSEA software (http://www.gsea-msigdb.org/gsea/index.jsp). Sankey diagram was conducted to visualize the correlation between cuproptosis-related lncRNAs, mRNAs, and risk factors (protective/risk) using the R package “ggalluvival”.

### Statistical analysis

All statistical analyses were conducted in the R software (Version 4.1.1). Student’s t-tests were applied to determine the difference between the two groups. For the analysis of differences between K-M curves, the log-rank test was performed. If there is no special description for the above method, statistical significance is defined as *p*-value < 0.05.

## Results

### Data of patients with skin cutaneous melanoma

**FIGURE 1:**
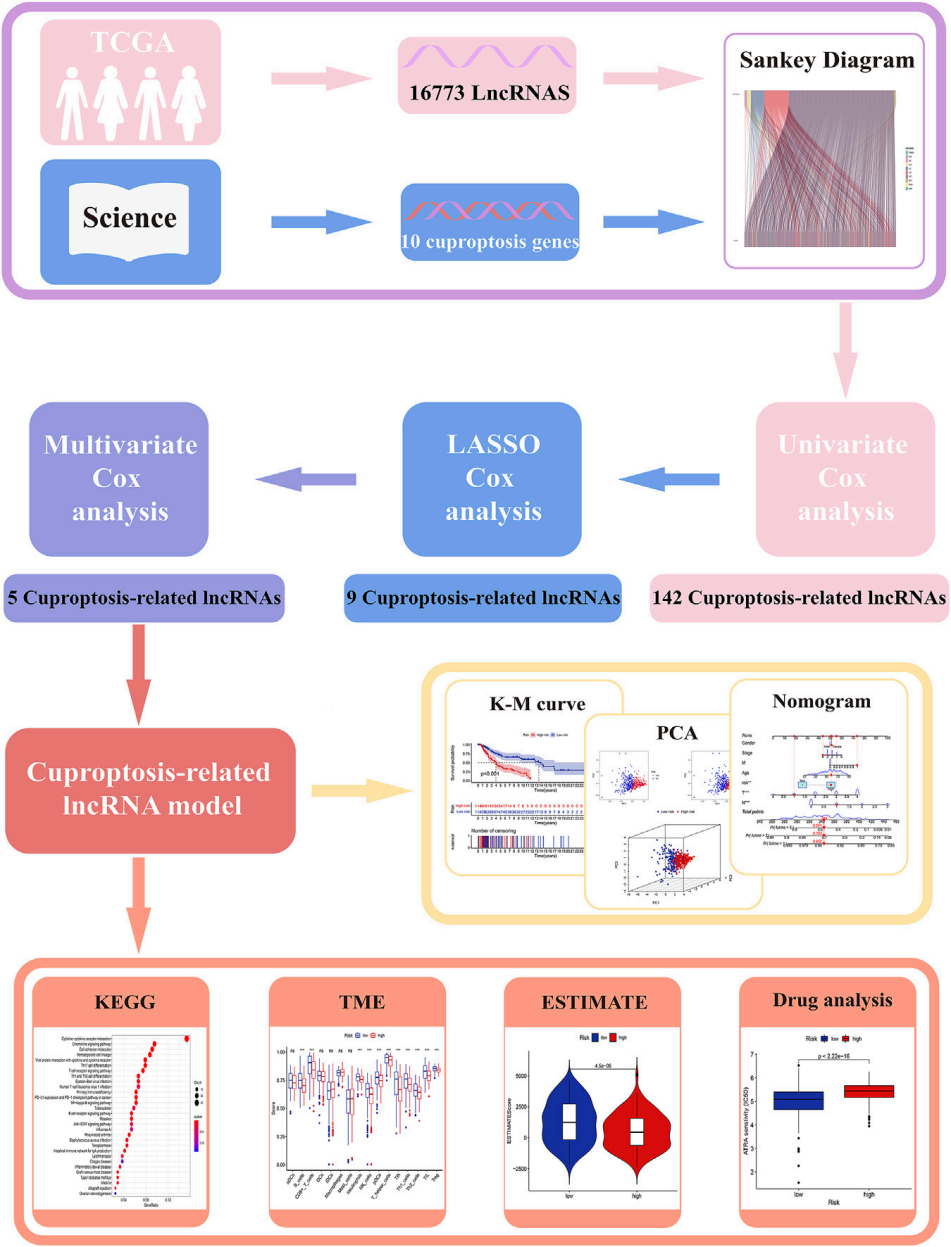
The workflow of this study.

All analysis processes were presented in the flow chart (Figure 1). In total, 455 patients with SKCM were considered in the subsequent study. Training set including 228 SKCM patients was applied to identify cuproptosis-related lncRNAs related to prognosis and further construct the prognosis risk model, and testing set including 227 SKCM patients was applied to verify the superiority of the established risk model. It has been found that clinical characteristics such as age, gender, and TNM stage are not statistically different between the two groups (Supplementary Table S2, *p* > 0.05).

#### Acquisition of cuproptosis-related lncRNA

**FIGURE 2:**
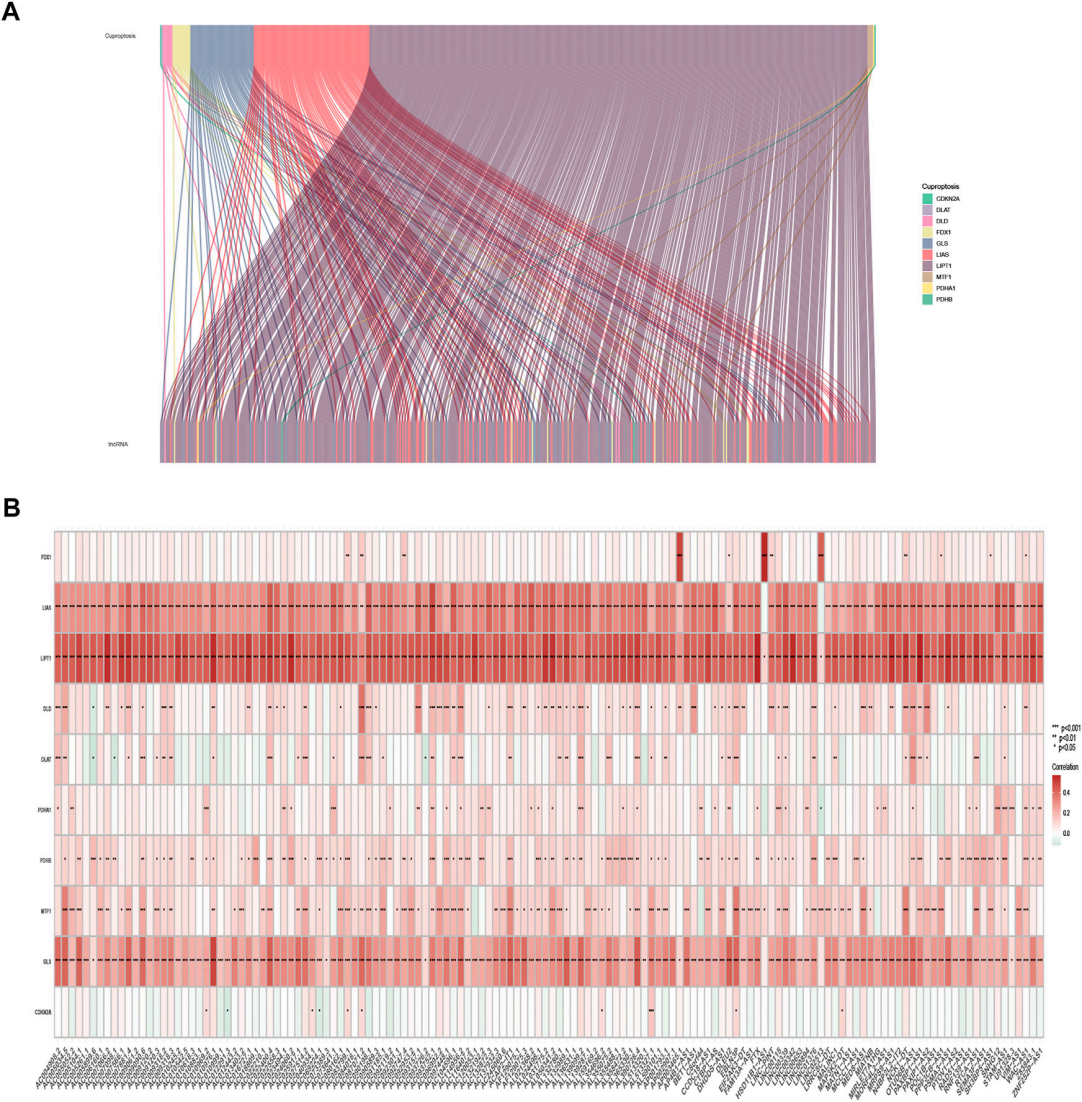
Identification of cuproptosis-related lncRNAs in TCGA-SKCM patients. **(A)** Co-expression network in Sankey diagram for cuproptosis-associated genes and corresponding lncRNAs. **(B)** The heatmap of 10 cuproptosis-associated genes and 142 cuproptosis-related lncRNAs.

The expression of 10 cuproptosis-associated genes and 16773 lncRNAs was identified from the TCGA database. After Pearson correlation analysis with filter criteria (|R| ≥ 0.4 and *p* < 0.001), we obtained 437 cuproptosis-related lncRNAs (Supplementary Table S3). The co-expression network between 10 cuproptosis-associated genes and 437 cuproptosis-related lncRNAs was presented in Figure 2A.

### Construction of the cuproptosis-related lncRNA risk model for skin cutaneous melanoma

**FIGURE 3:**
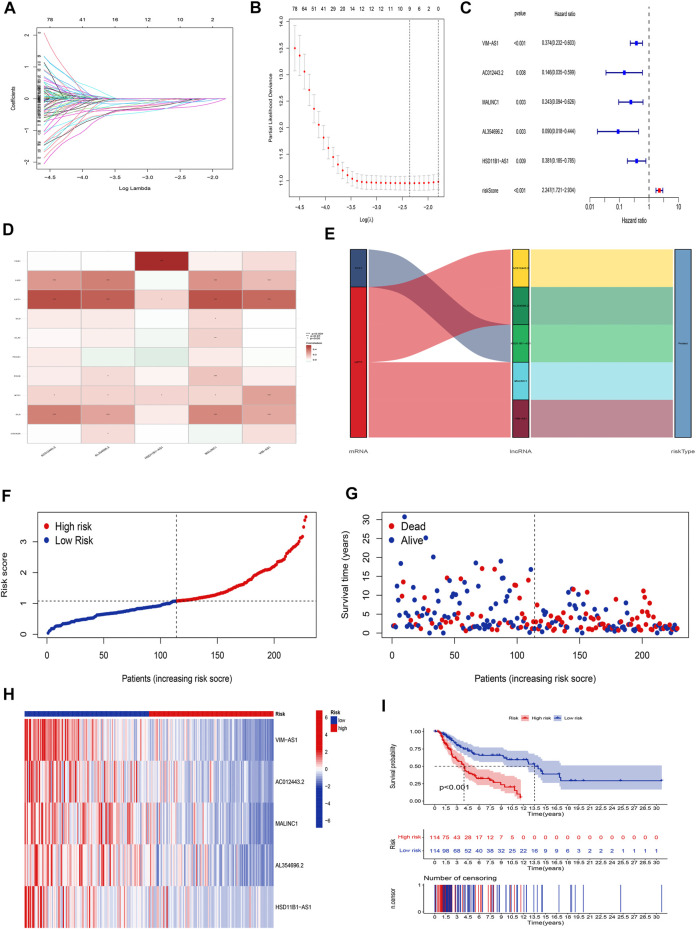
Construction of the cuproptosis-related lncRNA risk model. **(A,B)** LASSO regression analysis identified 9 cuproptosis-related lncRNAs. **(C)** Multivariate Cox regression analysis identified 5 cuproptosis-related lncRNAs. **(D)** Heatmap of the correlation between hub lncRNAs and cuproptosis mRNAs **(E)** The Sankey diagram shows the connection degree between cuproptosis mRNAs, cuproptosis-related lncRNAs, and risk types. **(F)** Distribution of risk scores, **(G)** survival status and survival time patterns, **(H)** relative expression of 5 hub lncRNAs, and **(I)** K-M survival based on the training set.

The univariate Cox regression analysis was applied to identify cuproptosis-related lncRNAs associated with OS, and 142 lncRNAs were identified (Supplementary Table S4). The correlation heatmap between 142 candidate lncRNAs and cuproptosis-associated genes was visualized in Figure 2B. Furthermore, the LASSO regression was applied and we found that 9 cuproptosis-related lncRNAs were significantly correlated with the prognosis of SKCM patients (Figures 3A,B). Finally, 5 cuproptosis-related lncRNAs, including VIM-AS1, AC012443.2, MALINC1, AL354696.2, and HSD11B1-AS1 were identified to construct the risk model using multivariate Cox regression analysis (Figure 3C; Table 1). The risk score was calculated based on the corresponding Cox regression model coefficients and lncRNA expression levels: risk score = VIM-AS1 × -0.486200693258448 + AC012443.2 × -1.12449989484336 + MALINC1 × -0.696789508906321 + AL354696.2 × -1.17823680822397 + HSD11B1-AS1 × -0.623186481172742.

**TABLE 1:** The 5 cuproptosis-related prognostic lncRNAs.

Id	coef	HR	HR.95L	HR.95H	*p*-value
VIM-AS1	−0.486200693	0.374195336	0.232070722	0.60335982	0.0000551
AC012443.2	−1.124499895	0.145536422	0.035367734	0.598874959	0.00757806
MALINC1	−0.696789509	0.243172712	0.094413127	0.626321461	0.003397614
AL354696.2	−1.178236808	0.089929911	0.018226757	0.443709709	0.00309874
HSD11B1-AS1	−0.623186481	0.381308182	0.185156678	0.785258902	0.008900943

We applied correlation test to further explore the co-expression network between 5 cuproptosis-related lncRNAs and mRNAs (Figure 3D). Besides, the Sankey diagram revealed that 5 hub cuproptosis-related lncRNAs were protective factors (Figure 3E). We investigated the distribution of risk scores in survival time and survival status between high- and low-risk groups in the training set (Figures 3F,G). Besides, the relative expression of 5 hub cuproptosis-related lncRNAs was calculated in the training set (Figure 3H). Compared with patients in the high-risk group, patients in the low-risk group have higher expression levels of 5 cuproptosis-related lncRNAs, which was consistent with the Sankey diagram. Finally, we applied the K-M curve to verify whether there was a significant difference in OS between high- and low-risk groups. The results showed that in the low-risk groups, SKCM patients had better OS compared with the high-risk groups (Figure 3I, *p* <0.001).

### Validation of the cuproptosis-related lncRNA risk model

**FIGURE 4:**
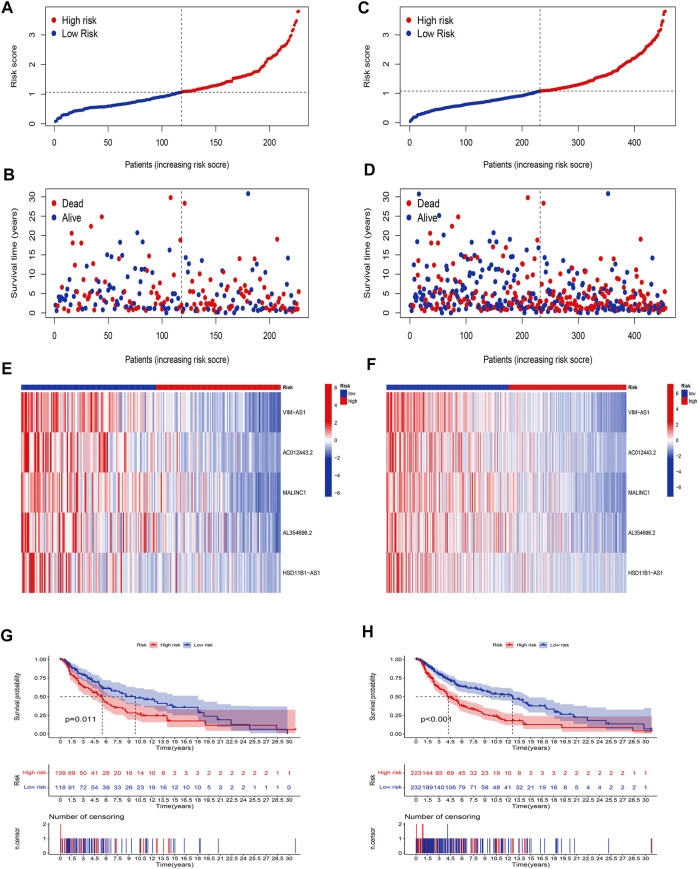
Validation of the risk model. Distribution of risk scores based on the testing set **(A)**, and entire set **(B)**. Survival status and survival time patterns are based on the testing set **(C)**, and the entire set **(D)**. Relative expression of 5 hub lncRNA based on the testing set **(E)**, and entire set **(F)**. K-M analyses are based on the testing set **(G)**, and the entire set **(H)**.

Our next step was to apply the testing set as well as the entire set to test the reliability of the established risk model. Using the method mentioned before, the risk curve as well as scatters plot to visualize the survival status and survival time suggested that SKCM patients in the low-risk group had longer survival time and a lower risk score than in the high-risk group, based on the results of the testing set (Figures 4A,B) and the entire set (Figures 4C,D). Furthermore, the heatmap of expression levels based on the testing set (Figure 4E) and the entire set (Figure 4F) confirmed that 5 hub cuproptosis-related lncRNAs were protective factors. K-M analyses also presented that low-risk SKCM patients had better overall survival than high-risk patients based on the testing set (Figure 4G, *p* <0.001) and the entire set (Figure 4H, *p* <0.001). The above bioinformatics studies fully identify that our established risk model has reliable discrimination for SKCM patients.

### PCA and *t*-SNE

**FIGURE 5:**
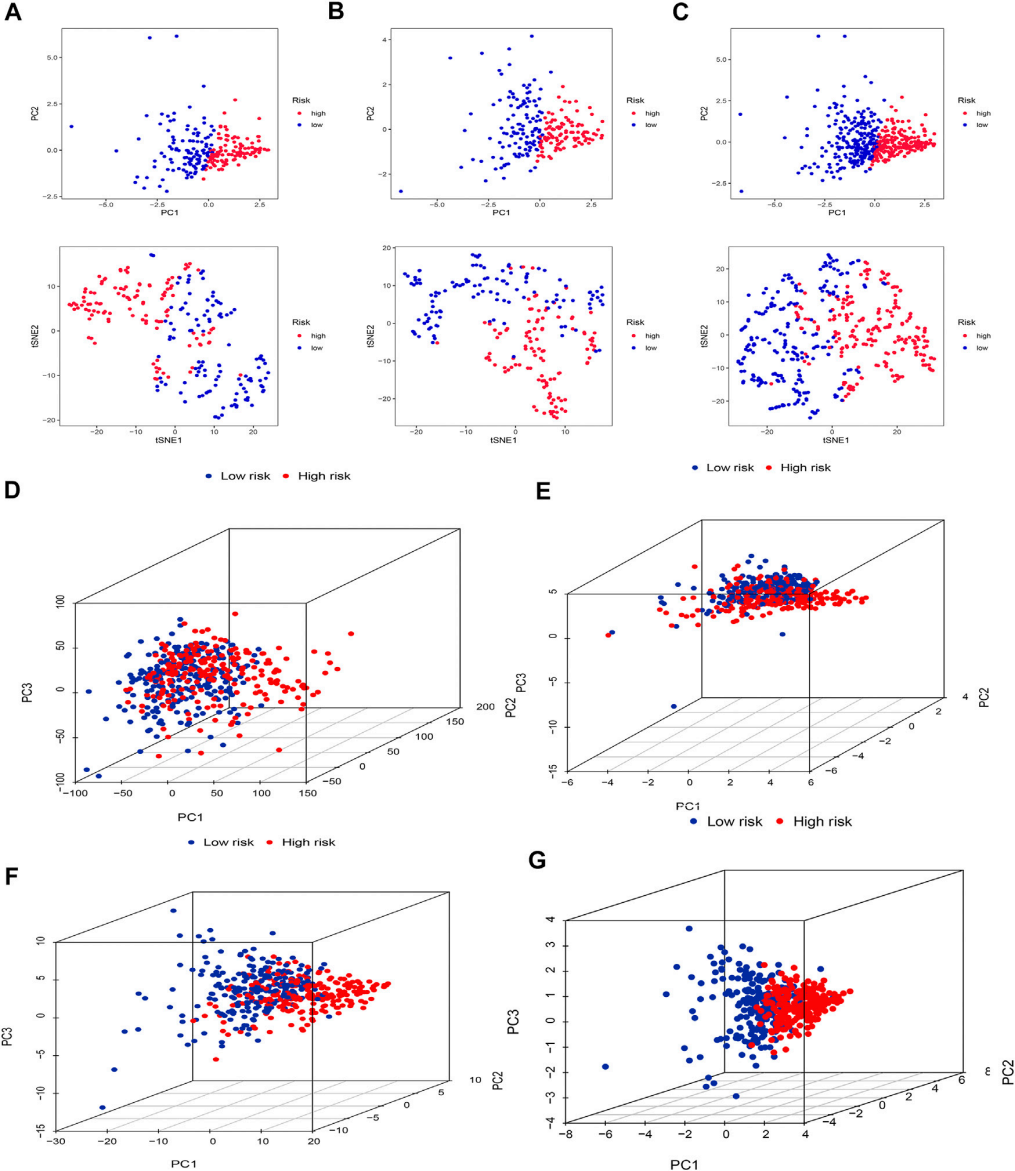
PCA and *t*-SNE. PCA and *t*-SNE analyses between the high-risk and low-risk groups based on the training set **(A)**, testing set **(B)**, and entire set **(C)**. PCA analysis between the high-risk and low-risk groups based on all entire gene expression profiles **(D)**, cuproptosis genes **(E)**, 437 cuproptosis-related lncRNAs **(F)**, and risk model **(G)**.

Firstly, we applied PCA and *t*-SNE analyses to evaluate the accuracy of the risk model based on the 5 cuproptosis-related lncRNAs in the training set (Figure 5A), testing set (Figure 5B), and entire set (Figure 5C). All results presented fairly significant discrimination between the two subgroups. Furthermore, we applied PCA based on the entire gene sequencing data of the TCGA-SKCM cohort, 10 cuproptosis-associated genes, 437 cuproptosis-related lncRNAs, and the risk prognostic model (Figures 5D–G). The distribution of the two groups based on the risk model was significantly different and stable, which fully indicated that the risk model can accurately distinguish SKCM patients and reflected the significant differences in the cuproptosis sensitivity between the two subgroups.

### Independent factor test and creation of nomogram

**FIGURE 6:**
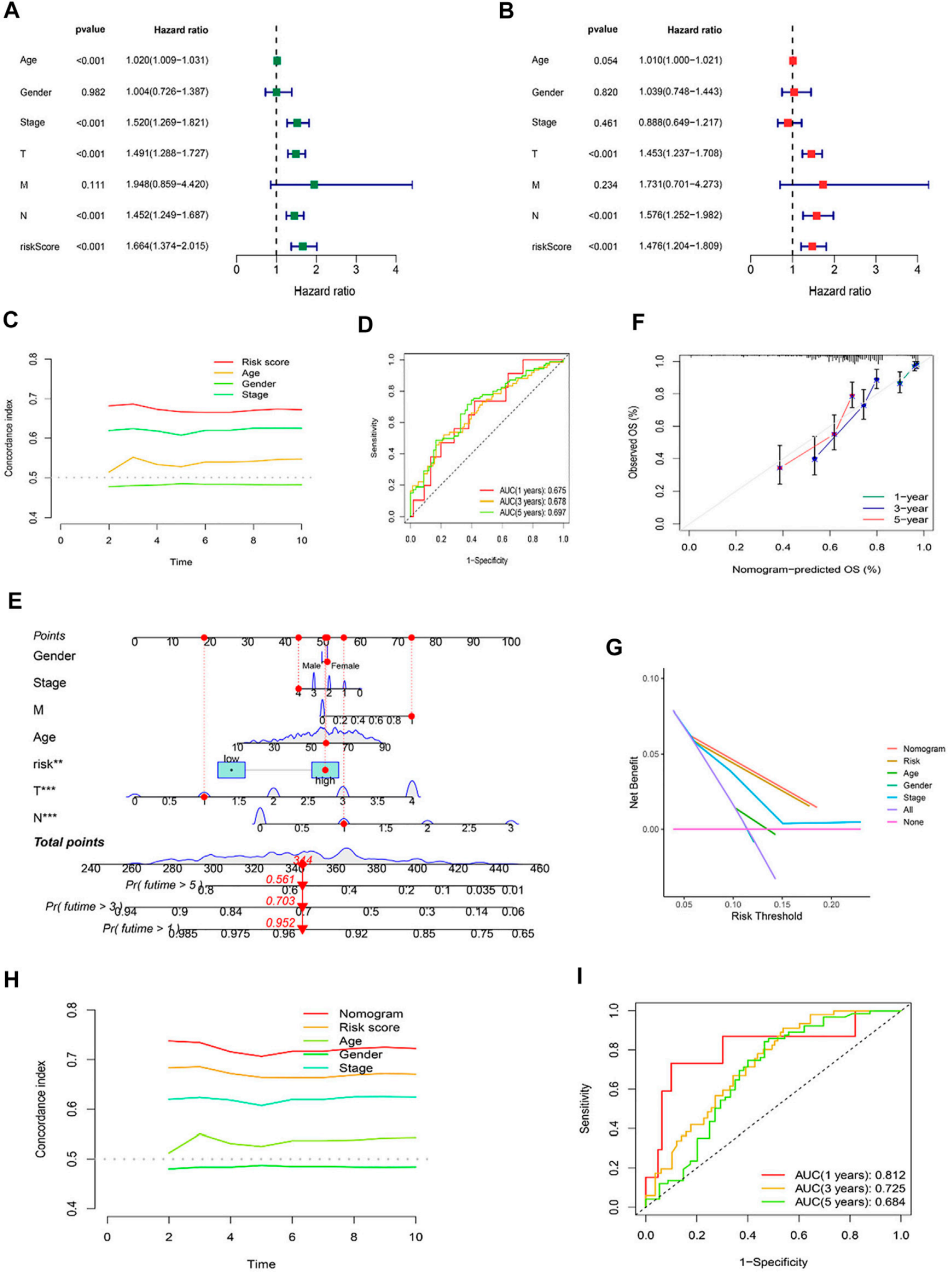
Independent Prognostic Factors and Construction of Nomogram. Forrest plot of the univariate Cox regression analysis **(A)**, and multivariate Cox regression analysis **(B)**, based on the entire set. **(C)** The concordance index of risk score with clinical characteristics. **(D)** ROC curves for 1-, 3-, and 5-years OS in the risk model. **(E)** The nomogram. **(F)** The calibration curves of the nomogram predict the probability of the OS (The x-axis shows nomogram-predicted survival, and the y-axis shows actual survival. The grey line shows the ideal calibration line, and the color line represents the model-predicted calibration line. **(G)** The DCA curves of the nomogram. **(H)** The concordance index of nomogram, risk score and clinical characteristics. **(I)** ROC curves for 1-, 3-, and 5-years OS in the nomogram.

Using univariate and multivariate Cox regression analyses to determine whether the cuproptosis-related lncRNA was an independent prognostic factor for OS in SKCM patients, we examined the potential independent effect of cuproptosis-related lncRNAs on our outcomes. The results of univariate Cox regression analysis suggested that age (HR = 1.020, *p* < 0.001), stage (HR = 1.520, *p* < 0.001), T stage (HR = 1.491, *p* < 0.001), N stage (HR = 1.452, *p* < 0.001), and risk score (HR = 1.664, *p* < 0.001) were significantly associated with OS (Figure 6A). After adjusting for other confounding factors, the multivariate Cox regression analysis showed that the risk score (HR = 1.476, *p* < 0.001) still had a significant effect on survival and prognosis (Figure 6B). Based on the above results, it is concluded that the risk prognostic model according to five cuproptosis-related lncRNAs serves as independent prognostic factors for SKCM patients. Besides, compared with other clinical indicators, the risk model showed the highest C-index (Figure 6C), and its AUC for 1-, 3-, and 5-years OS were all greater than 0.5, indicating the reliability of the model (Figure 6D). Considering the widespread use of nomogram and risk scores has an excellent ability to predict the prognosis of SKCM patients. We further constructed a nomogram by integrating multiple clinical factors and our constructed risk score to better predict 1-, 3-, and 5-years survival in SKCM patients (Figure 6E). The accuracy of the nomogram was verified in subsequent calibration curves, and we found a high degree of accuracy between the actual observed and predicted values (Figure 6F). Furthermore, the DCA curves based on the entire set also confirmed the superior predictive power of the nomogram (Figure 6G), and the nomogram even shows a higher C-index than the risk model (Figure 6H). Besides, the area under the ROC curve (AUCs) at 1, 3, 5-years were 0.812, 0.725, and 0.684, suggesting that the nomogram was reliable in predicting the OS of SKCM patients (Figure 6I).

**FIGURE 7:**
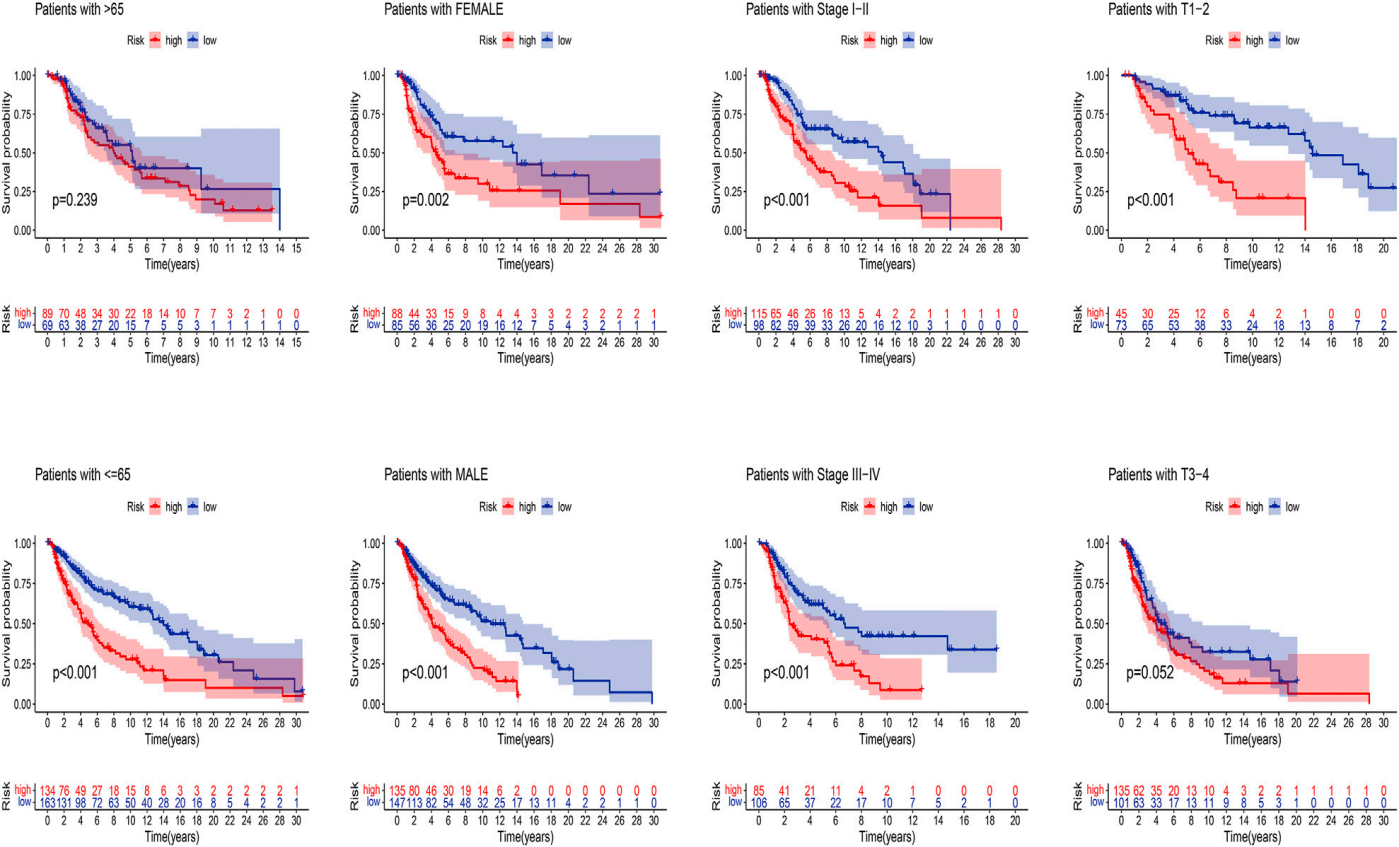
K-M analysis of OS stratified by age (≤65 or >65), gender (female or male), SKCM stage (I–II or III-IV), and TNM stage (T1–2 or T3–4) between high-risk and low-risk groups in TCGA entire set.

Next, we applied K-M analysis to verify whether our constructed risk model still maintains superior predictive power in different clinical traits. We found that high-risk patients of SKCM still had a lower prognosis in different groups of clinical features such as age, gender, pathological stage, such as TNM stage (Figure 7).

### Analysis of immune infiltration landscape

**FIGURE 8:**
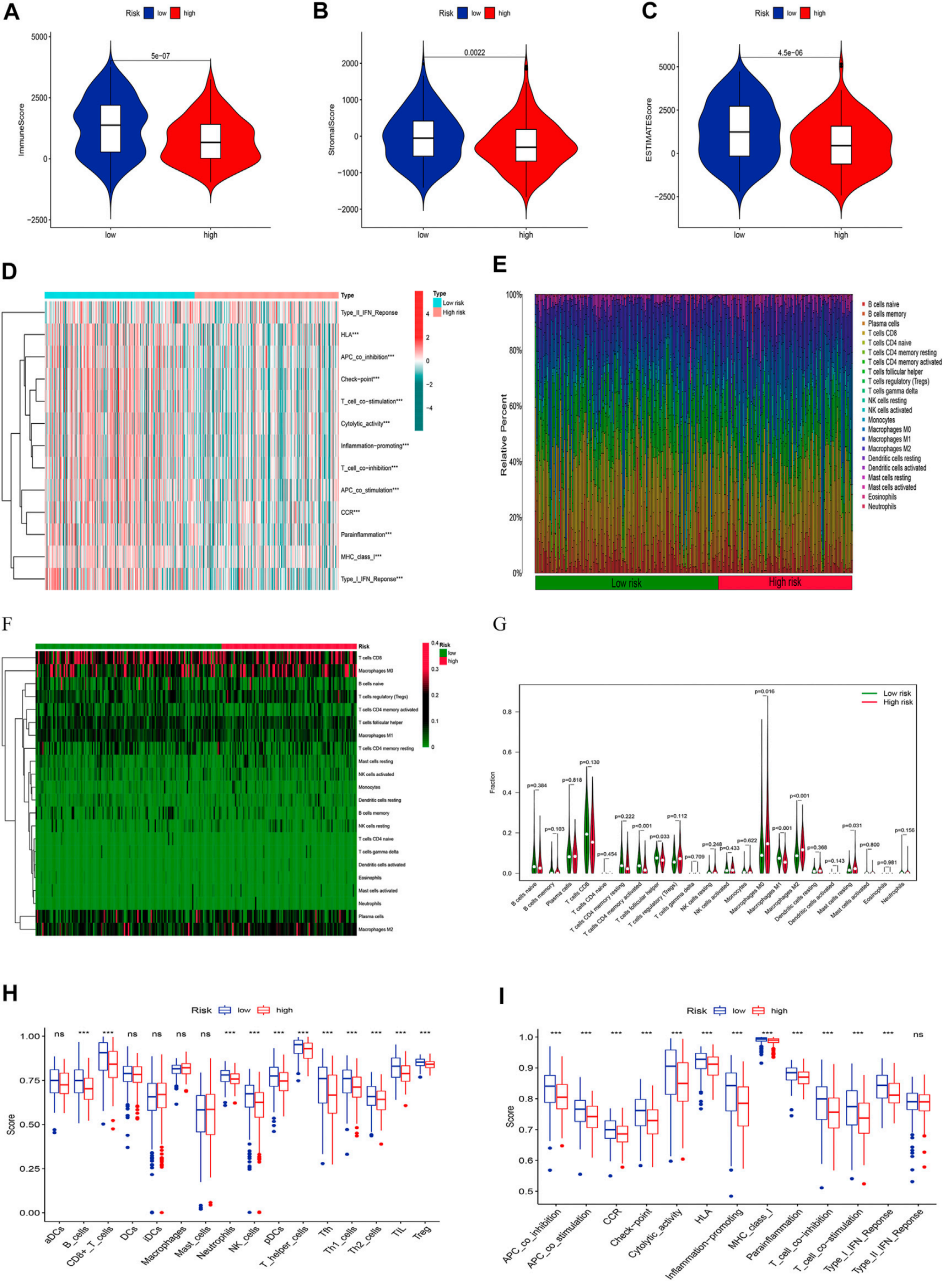
Analyses of tumor immune microenvironments between two groups. The differences of the immune score **(A)**, stromal score **(B)**, and estimate score **(C)**. **(D)** The GSVA of immune-related pathways between two groups. Expression features of 22 immune cells in the box plot **(E)**, heatmap **(F)**, and violin plot **(G)** using the CIBERSORT algorithm. The differences of the immune cells **(H)**, and immune functions **(I)** using the ssGSEA algorithm.

Given the importance of TME in tumor progression and treatment, we applied multiple immune assessment algorithms to study it. First, we applied the ESTIMATE algorithm to analyze the immune, stromal, and estimate scores of SKCM patients. It was found that SKCM patients in the low-risk group had a significantly higher immune score, stromal score, and estimate score than in the high-risk group (Figures 8A–C, *p* <0.05). Besides, the GSVA enrichment analysis revealed that SKCM patients in the low-risk group were significantly related to immune pathways and functions such as Type_II_IFN_Reponse, HLA, APC_co_inhibition, Check−point, Cytolytic_activity, and CCR (Figure 8D). We further performed the CIBERSORT algorithm to analyze the category and proportion of 22 immune cells. The relative fraction of 22 immune cells within low- and high-risk groups were presented by a box plot (Figure 8E), and the heatmap (Figure 8F) revealed significant disparities in the distribution of immune cells based on the risk model. Besides, compared to the high-risk group, we summarized that Macrophages M0, M2, and Mast cells resting account for a small proportion in the low-risk group (*p* < 0.05), while T cells CD4 memory activated, T cells follicular helper, and Macrophages M1 cover a larger proportion (*p* < 0.05) (Figure 8G). Finally, we further applied the ssGSEA algorithm to investigate the infiltration of immune cells and immune functions in the high-risk and low-risk groups. The results presented that the immune cells subpopulations of B cells, CD8^+^ T cells, Neutrophils, NK cells, (plasmacytoid DCs) pDCs, T helper cells, Tfh, Th1 cells, Th2 cells, TIL, and (regulatory T cells) Tregs were significantly higher in the low-risk group (Figure 8H, *p* <0.001). The APC co-inhibition/stimulation, chemokine receptors (CCR), Check-point, Cytolytic activity, human leukocyte antigen (HLA), Inflammation−promoting, MHC class I, Parainflammation, T-cells co-inhibition/stimulation, and type I IFN response was significantly upregulated in the low-risk group (Figure 8I, *p* <0.001). Finally, to assess the correlation between the risk score and immune cell subtype infiltration, we conducted a comprehensive analysis using multiple algorithms including TIMER, CIBERSORT, xCELL, quanTIseq, MCPcounter, EPIC, and CIBERSORT-ABS (Supplementary Figure S1). The results indicated that there was a negative relationship between immune cell infiltration and risk score. As shown above, the low-risk group had a higher level of immune infiltration, which may be associated with a better prognosis.

### Somatic mutation analysis

**FIGURE 9:**
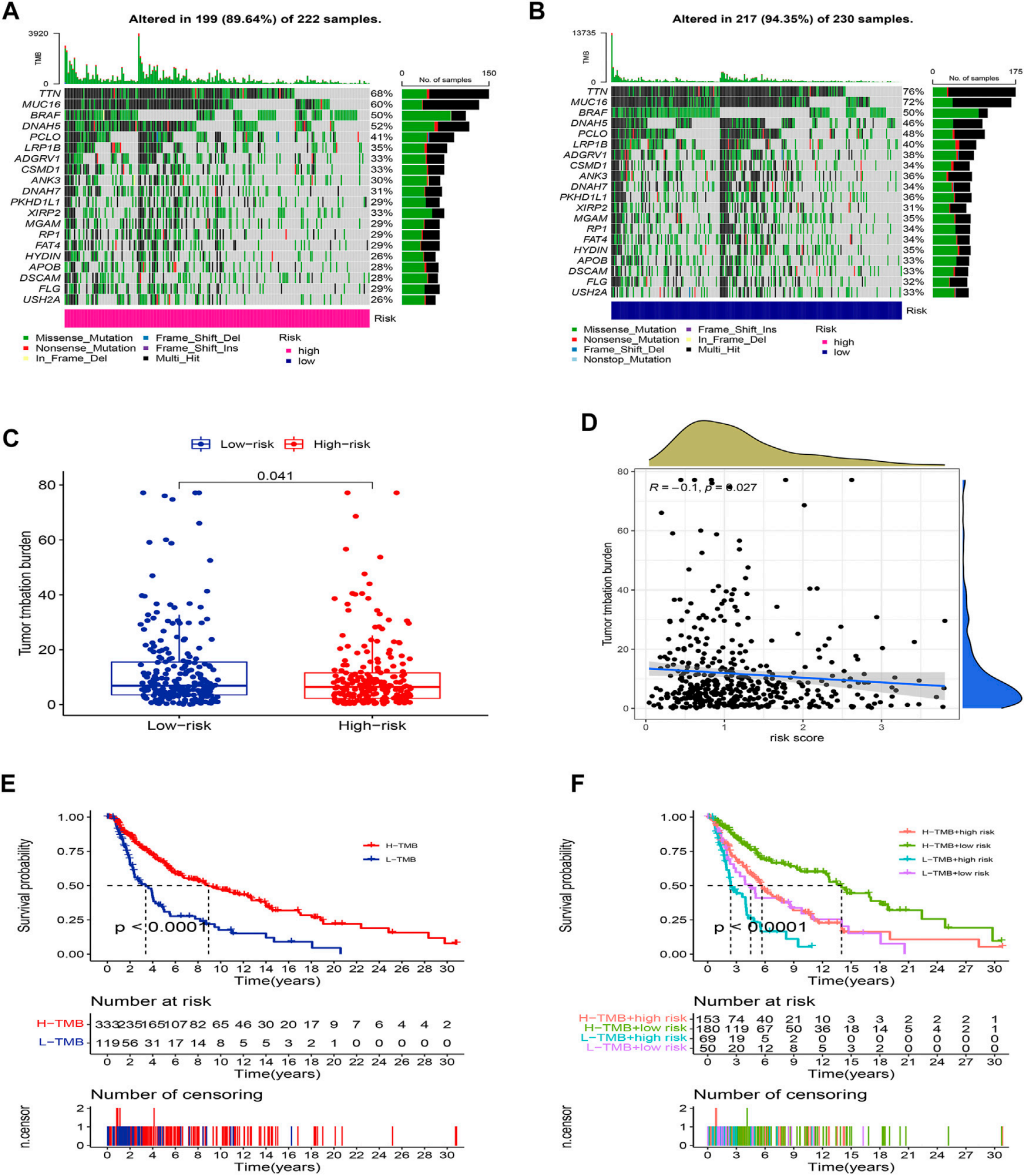
Landscape of mutation between two groups. The mutation distributions of patients in the high-risk group **(A)**, and low-risk group **(B)**. **(C)** The difference in TMB score between the two groups. **(D)** Correlations between risk score and TMB. **(E)** K-M analysis of the OS between high- and low-TMB groups. **(F)** K-M analysis of the OS between four groups stratified by both TMB and risk score.

We further compared the differences in the somatic mutations between the two groups. The low-risk group had a higher mutation rate (Altered in 217 (94.35%) of 230 samples) than the low-risk group (Altered in 199 (89.64%) of 222 samples), and the top 20 driver mutation genes were displayed in Figures 9A,B. Numerous studies already exist confirming that TMB can be a valuable predictor of tumor immune response and that patients with higher TMB might more benefit from immunotherapy (Hodi et al., 2021). The TMB quantitative analysis revealed that the low-risk group patients had a significantly higher TMB score compared to the high-risk group patients, suggesting that patients in the low-risk groups might be better candidates for immunotherapy (Figure 9C, *p* <0.05). We also conducted a Pearson correlation analysis to determine whether the risk model correlates with the TMB. The result suggested that there was a negative correlation among them (Figure 9D, *R* = -0.1, *p* <0.05). Then we divided all SKCM patients into high-TMB and low-TMB groups according to the cut-off values (media TMB score). Subsequently, K-M analysis suggested that patients in the high-TMB group had a significantly better OS than in the low-TMB group (Figure 9E, *p* <0.05). Using the TMB score to predict the survival of SKCM patients or using the risk model to predict the prognosis of patients, which one had the better predictive ability? Interestingly, when we combined the TMB and risk scores for K-M analysis of SKCM patients, we found that better OS with high-TMB was eliminated by the risk score. On the contrary, the patients in the group (low-risk score and high TMB score) had a significantly OS than in the other groups, and it could be concluded that the risk model was superior to the TMB in predicting an individual’s prognosis (Figure 9F, *p* <0.05).

### Functional enrichment analysis

**FIGURE 10:**
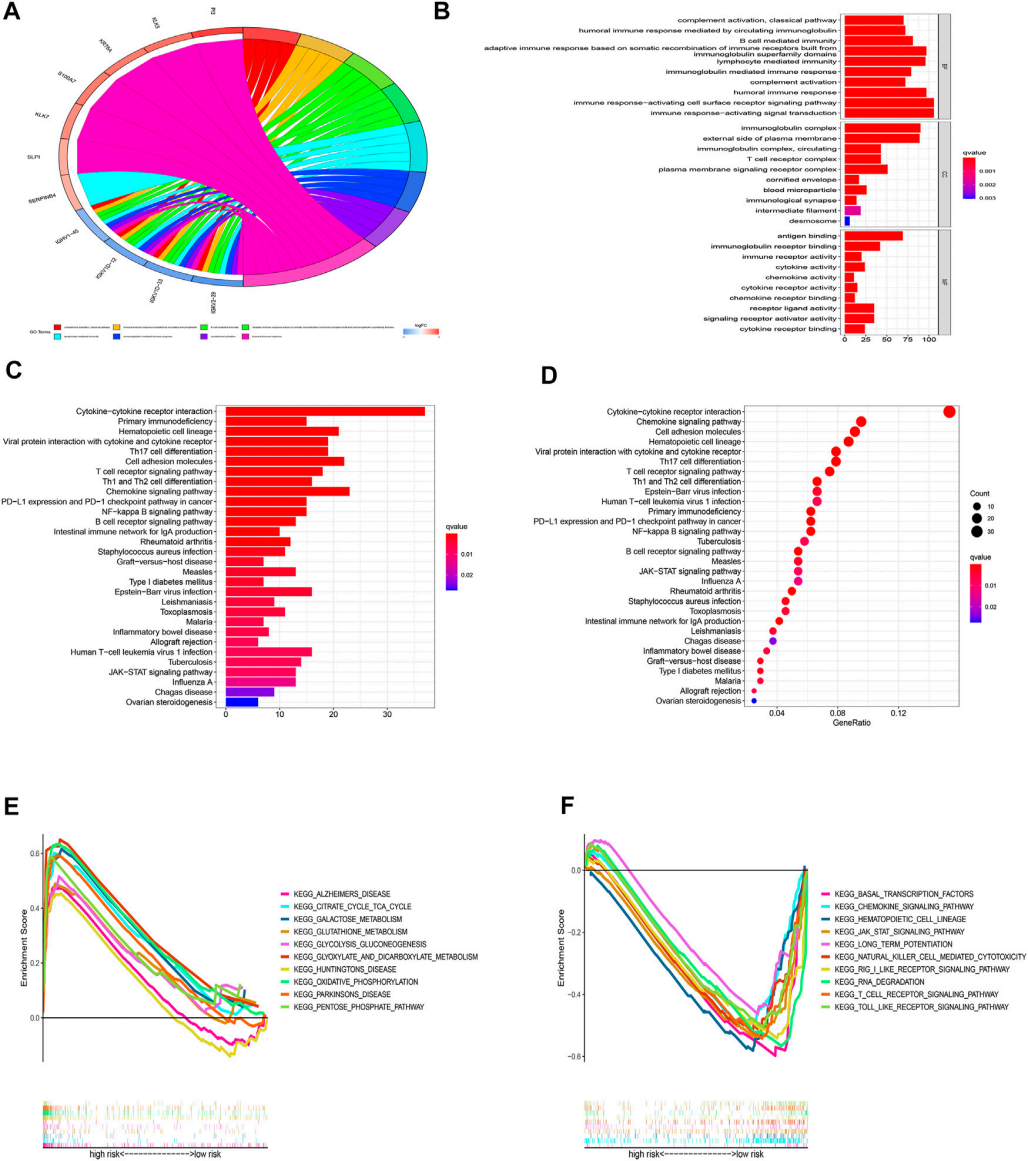
Functional enrichment analyses. **(A-B)** GO analysis based on DEGs between high- and low-risk groups. **(C-D)** KEGG analysis based on DEGs between high- and low-risk groups. **(E-F)** GSEA enrichment analysis.

For a deeper exploration of the mechanisms that contribute to significant differences between two groups in the multidimensional analysis. We further performed the GO and KEGG analyses (Supplementary Table S5) based on the 749 DEGs (Supplementary Table S6) between two groups (|Log2FC| > 1.0, *p-*value < 0.05). As displayed in Figures 10A,B, According to GO analysis, DEGs were significantly enriched in immune-related biological processes (BP), including lymphocyte-mediated immunity, immune response-activating cell surface receptor signaling pathways, immune response-activating signal transduction pathways, and humoral immune responses. In regards to cellular component (CC), these DEGs were significantly enriched in immunoglobulin complex and the external side of the plasma membrane. In regards to molecular function (MF), these DEGs were significantly enriched in antigen binding and immunoglobulin receptor binding. Besides, the KEGG analysis also indicated that these DEGs were mainly enriched in immune-related pathways such as Cytokine−cytokine receptor interaction, Primary immunodeficiency, Chemokine signaling pathway, and PD−L1 expression and PD−1 checkpoint pathway in cancer (Figures 10C,D). Additionally, the GSEA analysis of KEGG indicated that pathways such as glutathione metabolism, galactose metabolism, and oxidative phosphorylation were enriched in the high-risk group (Figure 10E; Supplementary Table S7 **)**, while such as the jak stat signaling pathway, chemokine signaling pathway, and other immune-related pathways were enriched in the low-risk group (Figure 10F; Supplementary Table S7). The results indicated that cuproptosis may be closely related to metabolism and immunity.

### Analysis of drug and immunotherapy response

**FIGURE 11:**
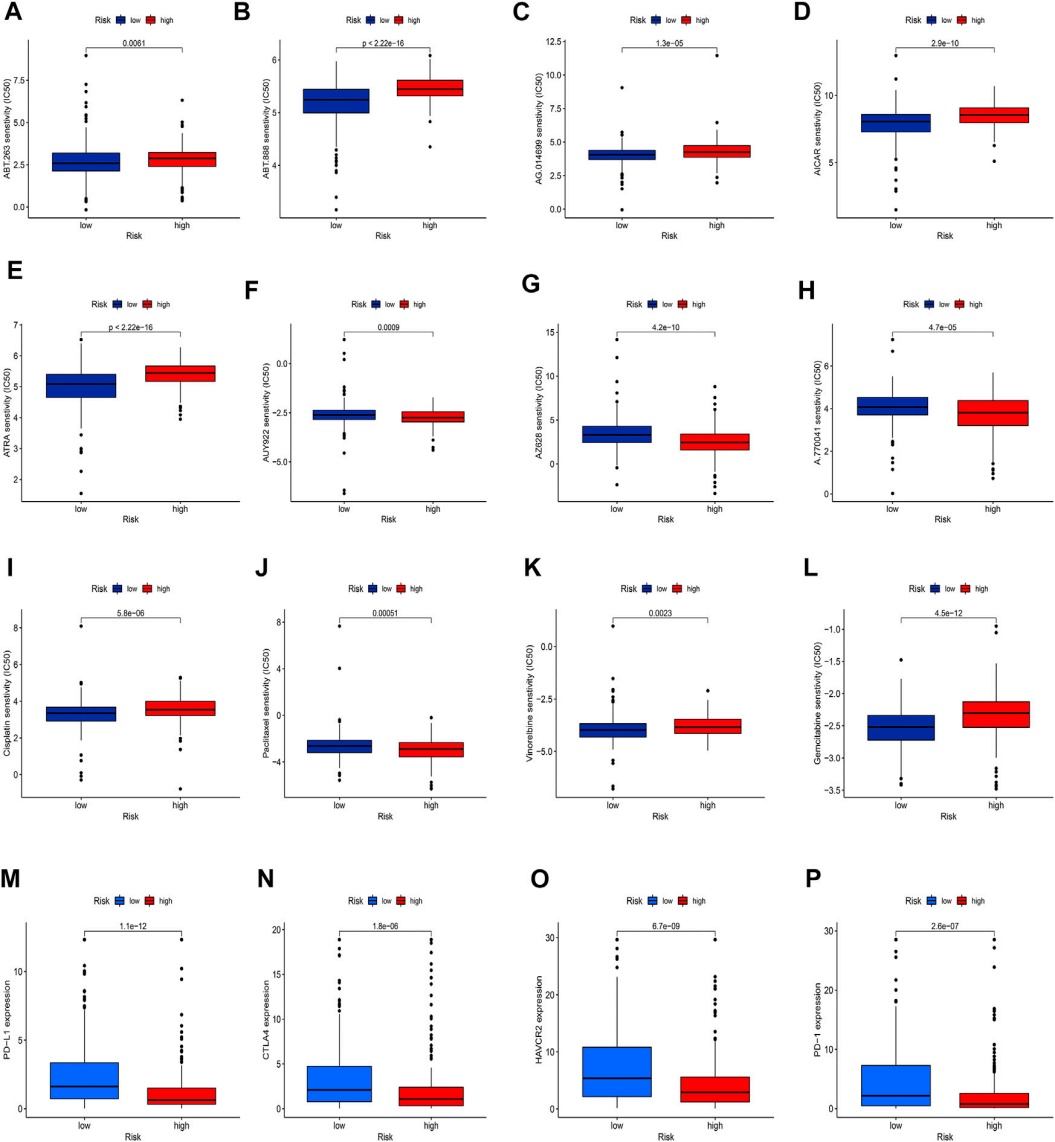
Exploration of therapeutic sensitivity. **(A–H)** Analysis of potential drug sensitivity in two groups. **(I–L)** Analysis of common chemotherapeutic sensitivity. **(M–P)** Expression levels of critical ICIs in the two groups. (*p* < 0.05 *; *p* < 0.01 **; *p* < 0.001 ***).

Given the significantly different prognosis of SKCM patients in two groups, we decided to further screen potential drugs to better achieve targeted therapy. We applied the R package “pRRophetic” to investigate the treatment response according to the IC50 values of samples in the GDSC database. According to the potential drugs analysis, we found that the IC50 values of 5 potential drugs (ABT.263, ABT.888, AG.014699, AICAR, ATRA) were significantly higher in the high-risk group, indicating that patients in the low-risk group may be more suitable for these drugs (Figures 11A–E, *p* <0.05). By contrast, the IC50 values of 3 potential drugs (A.770041, AZ628, AUY922) were significantly upregulated in the low-risk group, suggesting that patients of SKCM in the high-risk group may be more suitable for these drugs (Figures 11F–H, *p* <0.05). Furthermore, we calculated the IC50 values of common anti-tumor drugs among two groups and we were surprised to find that the IC50 values of these chemotherapy drugs such as cisplatin, paclitaxel, vinorelbine, and gemcitabine were significantly higher in the high-risk groups, suggesting that the risk model can well guide individualized clinical treatment and assess the patient’s immune response (Figures 11I–11L, *p* <0.05). As more and more ICIs have been proven to be effective in cancer treatment in recent years, we further evaluated the expression of PD-1, PD-L1, CTLA4, and HAVCR2. We found that patients of SKCM in the low-risk group had significantly higher expression of PD-1, CTLA4, HAVCR2, and PD-L1 (Figures 11M–11P, *p* <0.05), which indicates that the risk models can serve as promising predictors for the use of immune checkpoint inhibitors.

## Discussion

Although much progress has been made in the screening, diagnosis, and treatment of SKCM, the prognosis of advanced malignant melanoma is still low (Hayward et al., 2017). Considering that the main reasons for this poor prognosis and high mortality are the lack of early and effective diagnostic tools and the early metastasis properties, we decide to use bioinformatics analysis to find effective biomarkers for early detection. In recent years, PCD has been regarded as one of the most promising anti-tumor mechanisms and was found to play a crucial role in regulating the progression of various cancers (Koren and Fuchs, 2021; Qi et al., 2022b). Additionally, recent studies point to a completely new PCD: copper-dependent programmed cell death named cuproptosis (Tsvetkov et al., 2022). Copper is an essential component of many biochemical reactions and is widely involved in a variety of cellular functions, such as cell metabolism, growth, and proliferation, protein activity regulation, as well as apoptosis, autophagy, and other cellular processes (Polishchuk et al., 2019; Yang et al., 2021). Several studies over the past few years have shown that abnormal copper levels in cells or circulating blood are associated with tumor progression and prognosis in tumor patients. Fang et al. (2019) found that higher serum copper levels are associated with poorer prognosis in patients with liver cancer. Besides, Lopez et al. (2019) confirmed that antagonizing copper uptake by tumor cells, or chelating or inactivating copper in cells can effectively inhibit tumor progression which is a very promising treatment strategy for cancer. Meanwhile, A growing body of research has revealed that lncRNAs play a crucial role in the biological process of SKCM as emerging genetic and molecular biomarkers (Wang et al., 2020; Guo et al., 2021). For example, Sun et al. (2021) established a novel ferroptosis-related lncRNA signature that can be used to predict the prognosis and provide immunotherapy treatment targets for SKCM. However, no studies have been conducted regarding the relationship between cuproptosis-related lncRNAs and SKCM. Based on the above research backgrounds, we have chosen to investigate how lncRNAs that can regulate the process of cuproptosis can impact tumor progression as well as patient outcomes in SKCM.

In our study, based on univariate Cox, LASSO, and multivariate Cox regression analyses, a total of five protective cuproptosis-related lncRNAs were identified to construct the risk prognostic model. It is worth mentioning that this step-by-step dimensionality reduction method to finally screen out genes that are key to prognosis and use it to build a risk model has been reported by many excellent articles and is reliable (Jiang et al., 2022b; Guo et al., 2022). The superior prognostic ability of the risk model was confirmed in subsequent research analyses. In addition, we created an accurate nomogram to better predict 1-, 3-, and 5-years survival rates in SKCM patients. We were surprised to discover that of the five cuproptosis-related lncRNAs (VIM-AS1, AC012443.2, MALINC1, AL354696.2, HSD11B1-AS1), except for AC012443.2 and AL354696.2, which have no related studies previously published, the other three were shown to be closely associated with cancer progression, especially SKCM. The lncRNA VIM-AS1 has been shown in numerous studies to regulate the growth and metastasis of various tumor cells. In addition, Xiong et al. (2021) reported that the lncRNA VIM-AS1 is found to have significantly high expression levels in metastatic bladder cancer tissues and that the VIM-AS1/miR-655/ZEB1 axis regulates the epithelial-mesenchymal transition in bladder cancer. Zhang et al. developed a risk model using immune-related lncRNA, including VIM-AS1, which provides insight into patients with lung adenocarcinoma prognosis (Zhang et al., 2021b). Additionally, Li et al. reported that lncRNA MALINC1 is also related to the immune response as well as prognosis of SKCM, and is applied to construct the risk prognostic model of SKCM (Li and Luo, 2021). This is consistent with our study, indicating that MALINC1 plays an important role in the progression of SKCM. It is worth noting that lncRNA HSD11B1-AS1 also proved to play a crucial role in the progress of SKCM (Liu et al., 2022) and breast cancer (Xu et al., 2021). Liu et al. confirmed that lncRNA HSD11B1-AS1served as a protective factor inhibits the proliferation, migration, and invasion of SKCM by multiple functional experiments (Liu et al., 2022). Our study found that lncRNAs AL354696.2 and AC012443.2 also act as protective factors to reduce the risk of SKCM and improve prognosis, but no relevant studies have reported them, so the subsequent mechanisms need to be further explored.

In the further analyses of immune infiltration, we found that both immune cells and immune functions presented higher infiltration status in low-risk group patients of SKCM, suggesting that the better prognosis of patients in the low-risk group may be associated with a higher immune infiltration status. Interestingly, we also noticed that the immune, stromal, and estimate scores were significantly upregulated in the low-risk group. These results suggest that the risk model we have developed is largely related to the immune landscape of the SKCM microenvironment. It also indicated that cuproptosis may mediate the expression of immune cells and immune function and thus affect tumor progression. Further investigation is necessary to identify the molecular mechanisms by which cuproptosis and SKCM immunity are linked. Numerous studies have shown that TMB can be used as a biomarker to predict the effectiveness of immunotherapy (Ricciuti et al., 2019; Wong et al., 2021). From previous studies, TMB has been shown to be highly correlated with clinical outcomes after immunotherapy in advanced melanoma, further suggesting a combined approach to assess TMB and inflammatory signatures that can well differentiate the response of patients with advanced melanoma to immunotherapy (Hodi et al., 2021). Interesting to note, that in our study, scores on the TMB test and gene mutations were significantly higher in the low-risk group than in the high-risk group. Higher TMB scores, better prognosis, and higher immune infiltration status in the low-risk group fully demonstrate that the risk model based on cuproptosis has better predictive value.

Due to the development of immunotherapies targeting CTLA4, PD-1, and PD-L1, treatment outcomes for SKCM patients have improved over the past decade. Although large-scale clinical studies using immune checkpoint-related drugs in SKCM are currently lacking, more and more studies have demonstrated the undeniable role of immune checkpoint-related genes in the progression of SKCM (Selitsky et al., 2019) (Zhong et al., 2022). To better estimate the efficacy of checkpoint blockade therapy on our risk model, we investigated the expression of critical ICPs and found that low-risk patients had higher expression levels. Here, we also evaluated the sensitivity of high-risk and low-risk patients to commonly used chemotherapeutic drugs to better guide clinical medication. Some potential compounds were also screened and may provide some new directions for the treatment of SKCM. We found that the risk model is a promising predictor for antitumor drug selection and provides reliable immune markers for tumor immunotherapy.

To better investigate the potential regulatory mechanisms of CM, we further explored the possible different signaling pathways between the two groups. GO and KEGG analyses indicated that the DEGs were largely associated with immune-related pathways. Among them, we found that in the functional enrichment, there are many T helper cell-related pathways (such as Th17, 1 and 2 cell differentiation, etc.). In addition, in the analysis of immune cell infiltration, we also found that the infiltration fraction of T helper cells was significantly different between the two different risk groups. The study of Tom Hartwig et al.(2018) pointed out the important role of Th cells in inhibiting skin inflammation and pointed out that the inhibitory effect of skin inflammation played by Th cells (mainly IL-17/17A secreted by them) is involved in psoriasis, melanin It plays an important regulatory role in cancer and other diseases. In addition, other studies have observed a decrease in the percentage of peripheral and tumor-infiltrating Th17 cells in SKCM patients (He et al., 2021), and a clinical study by Kyoko Yamaguchi et al. (Yamaguchi et al., 2018). These results are consistent with the finding that the low-risk group had a higher Th cell infiltration score in the immune infiltration analysis. However, we also noticed that the mechanism of action of Th17 cells in SKCM has not been thoroughly studied. The article by Chen and Gao. (2019) pointed out that Th17 cells performed like macrophages in tumors, which also have contradictory effects of promoting tumor and inhibiting tumor. Their summary indicates that the relationship between SKCM and Th cell infiltration requires more and more rigorous experiments to verify.

Here, we must also admit that there are some limitations in this study. First, this study is based on the analysis of SKCM samples from the TCGA database, and there may be sample bias in the analysis of a single database, resulting in one-sided analysis results; Literature studies, given that cuproptosis is a recently proposed concept, there may be genes that have not been discovered that have not been included in this study; finally, we need more experiments *in vivo* or *in vitro* to examine the lncRNAs for prognostic models building.

In conclusion, this study is the first to investigate the regulation of lncRNAs on the cuproptosis process of tumor cells in SKCM. Using the relevant data of SKCM patients in TCGA, we constructed a lncRNA prognosis prediction model based on the regulation of the cuproptosis process, which can shed the hoping light on the diagnosis and treatment of SKCM.

## Data Availability

The original contributions presented in the study are included in the article/Supplementary Material, further inquiries can be directed to the corresponding authors.
