# Supplementary figures and images for: Construction of five cuproptosis-related lncRNA signature for predicting prognosis and immune activity in skin cutaneous melanoma
Source: Front Genet. 2022 Sep 7;13:972899. doi: 10.3389/fgene.2022.972899 (PMC9490379; doi:10.3389/fgene.2022.972899)

Figure S1:

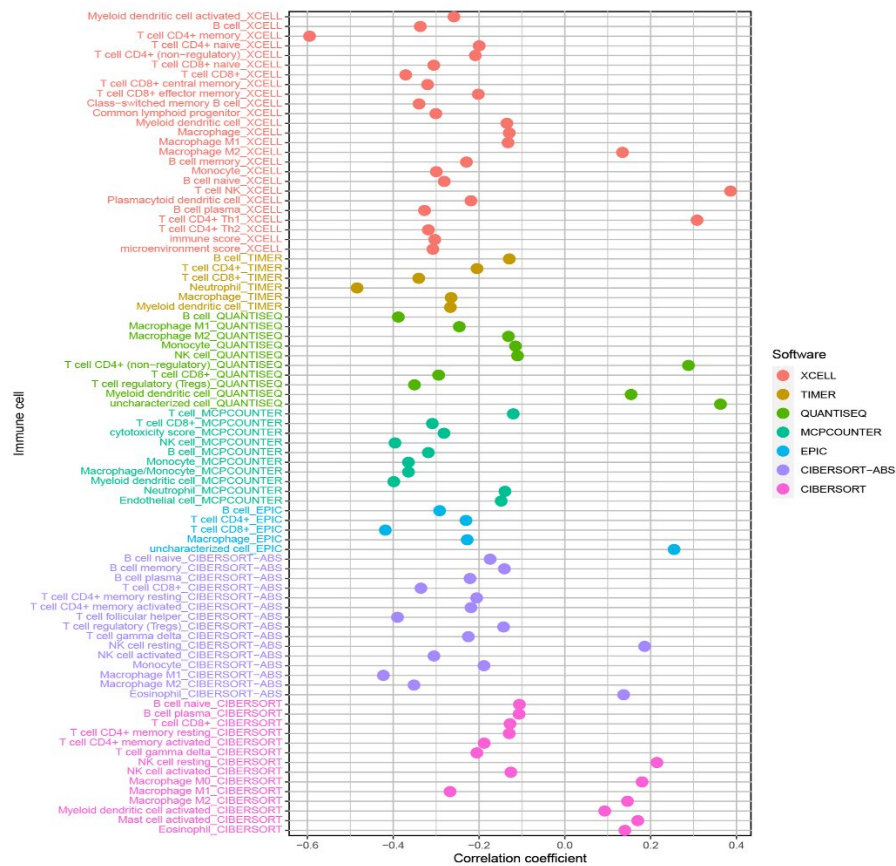

Figure S1| The relationship between the risk score and immune cells infiltration.

Supplement: Supplementary file 2 [file Image1.pdf]
